# Plant-Based Food: From Nutritional Value to Health Benefits

**DOI:** 10.3390/foods13223595

**Published:** 2024-11-11

**Authors:** Jiyuan Xue, Yongqi Yin

**Affiliations:** College of Food Science and Engineering, Yangzhou University, Yangzhou 225009, China; 18914539786@163.com

The United Nations’ 2030 Sustainable Development Goals present a transformative vision for addressing challenges related to food security, nutrition, and health, with plant-based foods poised to play a crucial role [1]. Throughout history, plant-based foods have been a significant component of human nutrition, and there has been a longstanding belief in the health benefits of consuming such foods [2,3]. Plant-based foods encompass a wide variety of fruits, vegetables, whole grains, and products derived from these ingredients. These foods not only provide essential proteins, vitamins, and minerals but are often rich in various phytochemicals, including polyphenols, flavonoids, isothiocyanates, carotenoids, alkaloids, and terpenes [4]. Numerous epidemiological studies have substantiated the rationale behind the interest in dietary phytochemicals, revealing a direct correlation between the consumption of plant-based foods and health outcomes [5]. Increased adherence to plant-based diets has been associated with a reduced incidence of metabolic syndrome and other chronic diseases, including cardiovascular conditions, certain types of cancer, neurodegenerative disorders, and obesity-related syndromes [6]. However, the diversity of plant species leads to significant variations in the distribution, concentration, composition, and bioavailability of health-promoting compounds and functional food components. Moreover, the biosynthesis of these bioactive substances within plants is also influenced by external environmental factors. Additionally, the processing techniques and formulations employed during the development of food products from these plant-based materials can significantly impact the concentration and composition of these bioactive components, thereby affecting the nutritional value of processed foods [7,8].

The primary focus of this Special Issue, titled “Plant-Based Food: From Nutritional Value to Health Benefits”, is to encompass the latest advancements related to plant-based foods, particularly concerning the impact of varieties or growth conditions on the bioactive compounds in plant materials, as well as strategies to maintain or enhance the nutritional quality of plant-based foods through modifications in processing techniques or product formulations. This lays the groundwork for the development and utilization of new plant resources and plant-based foods. This Special Issue presents 15 high-quality papers contributed by 14 research teams from 12 countries, including 13 original research articles and 2 review articles.

The role of plant biodiversity and growth environments in the variation of natural compounds in plants is well recognized. Describing the physicochemical properties of plants from different genotypes, cultivation regions, and growth stages, along with the identification and quantification of bioactive compounds and their bioactivity assessment, is essential for evaluating plant-based foods as sources of functional food or dietary supplement ingredients. Sweet potato (*Ipomoea batatas*), as the sixth largest food crop globally, is cultivated in over 100 countries and is a vital crop for ensuring food security in developing nations [9]. Lavhelani Tshilongo and his colleagues (contribution 1) compared the physiological and biochemical characteristics of leaves from five different genotypes of purple-fleshed sweet potatoes and identified bioactive substances within the leaves, while systematically investigating the variations in the content of these bioactive compounds throughout the entire cultivation cycle. They found that the genotype × harvest time × season interaction significantly influenced the total polyphenol content in the leaves (*p* < 0.001). The highest concentrations of four phenolic compounds and the greatest antioxidant scavenging activity were observed in the leaves of the South African native genotype (2 November 2019) of purple-fleshed sweet potato after eight weeks of cultivation. The specific harvest periods of South African native genotypes (2 November 2019 and Purple-purple) and the American imported genotype (08-21P) serve as excellent sources of anthocyanins and carotenoids. Their research delineated the optimal times for consuming purple-fleshed sweet potato leaves and utilizing them as functional ingredients, confirming the potential of sweet potato leaves to evolve into a sustainable plant-based food source.

Damask rose (*Rosa damascena* Mill.), a widely utilized aromatic industrial plant predominantly found in the Northern Hemisphere, encompasses approximately 200 species and 18,000 cultivated varieties [10]. Safoora Behnamnia et al. (contribution 2) employed LC-MS/MS to investigate the bioactive compounds in nine Iranian satin rose varieties. They identified several flavonoids, including phloridzin, diosmetin, and diosmin, in rose petals for the first time, noting significant variations in total flavonoid content among different varieties. Benzyl alcohol emerged as the primary aromatic compound in the tested rose varieties, with concentrations ranging from 69.28% to 77.58%. They concluded that the D234 variety, characterized by high yield, rich fragrance, and elevated total flavonoid content, holds considerable promise for diverse applications. This study provides a reference for the industrial cultivation and breeding of new varieties of Iranian satin rose. *Arctium lappa*, *Taraxacum officinale*, and *Melissa officinalis* are three representative edible and medicinal plants. Donatella Ambroselli and colleagues (contribution 3) conducted a comprehensive analysis of the metabolite profiles of these three crops grown in terrestrial, mountainous, and organic conditions using untargeted NMR-based metabolomics for the first time. The study revealed that soil, climate, and cultivation methods significantly influence the metabolite composition of these plants, with substances such as arginine and inositol exhibiting tissue specificity. This research establishes a foundation for the further development and utilization of the metabolite profiles of these three plants in the nutritional supplement, functional food, and phytopharmaceutical industries.

Microalgae have emerged as a promising alternative protein source and have entered large-scale commercial production in recent decades [11,12]. Their short growth cycles, strong resilience, and flexible cultivation methods, compared to traditional crops, make them a valuable new food ingredient with significant economic and environmental benefits, without occupying arable land resources. Currently, the investigation and application of microalgae in human diet represents an extremely promising yet still developing field. Natália Čmiková’s research team (contribution 4) explored the nutritional composition and physiological activity of five commercial microalgae. The protein content in these five microalgae is 34.09–42.45%. The minerals and essential amino acids in each microalga exhibit a unique distribution. *Tisochrysis lutea* is identified as a rich source of polyunsaturated fatty acids and is abundant in B vitamins such as niacin and riboflavin, along with a diverse array of polyphenolic compounds. The research confirmed that all microalgae exhibit high antioxidant activity. This study further elucidates the significant potential of microalgae as a source of bioactive compounds, indicating that further research and the exploration of the diverse properties of microalgae may yield creative and sustainable answers to global problems in resource use and nutrition.

Given that the bioactive components in plants are influenced by environmental conditions, recent years have seen an increasing number of studies artificially regulating plant growth environments to alter physiological traits, thereby directing the accumulation of bioactive substances within plants. Jing Zhang et al. (contribution 5) optimized the enrichment of phenolic compounds in wheat sprouts under LED illumination through response surface optimization experiments. Research has shown that red LED light promotes the synthesis of phenolic compounds by inducing the activity of key enzymes and their gene expression within the phenylpropanoid metabolic pathway, ultimately resulting in wheat sprouts that are rich in phenolic compounds and exhibit strong antioxidant capabilities. Mian Wang and colleagues (contribution 6) utilized UV-B radiation to regulate the biosynthesis of isoflavones in soybean suspension cells. According to their results, UV-B radiation increased the transcription levels and activity of important enzymes in soybeans that are involved in the manufacture of isoflavones, while the composition of isoflavones in the cells displayed notable tissue specificity. Xiaolan Quan et al. (contribution 7) explored the effects of exogenous sodium selenite treatment on the content of isothiocyanates and selenium in *broccoli* sprouts, employing proteomics to reveal the molecular mechanisms underlying the accumulation of bioactive substances. Their research demonstrated that sodium selenite treatment significantly upregulated the metabolic and biosynthetic pathways of secondary metabolites in *broccoli* sprouts. Collectively, these three studies confirm the feasibility of artificially regulating external environmental conditions to promote the accumulation of bioactive substances within plants, providing a foundational basis for the development of plant materials rich in bioactive compounds for nutritional supplements and dietary enhancements.

So far, the biological significance of bioactive compounds in plant-based foods has been widely acknowledged. In particular, phenolic and flavonoid compounds from various sources have demonstrated significant antioxidant, anticancer, and cardiovascular protective activities, supporting their application in the food and pharmaceutical industries [13]. Jin Cheng and colleagues (contribution 8) investigated the hepatoprotective effects of extracts from eight tea-like plants using an acute alcohol exposure model in C57BL/6J mice. Their research indicated that these eight tea-like plants could enhance the activity of alcohol-metabolizing enzymes, improve hepatic oxidative stress and inflammation, and modulate the gut microbiota, thereby providing varying degrees of liver protection. They identified several active compounds, including chlorogenic acid, epicatechin, rutin, and tannic acid, in the two tea-like plants that exhibited the most significant improvement in acute alcoholic liver injury (sweet tea and camellia). This study provides scientific evidence for the hepatoprotective effects of tea-like plants, encouraging the consumption of these teas or their development into functional foods for the prevention and treatment of alcohol-related liver damage, addressing the growing incidence of alcohol-related liver diseases. Aneta Szulc et al. (contribution 9) conducted a comprehensive review summarizing the main categories of flavonoids found in fruits, vegetables, and herbs, extensively describing the role of dietary flavonoids in the prevention and alleviation of neurodegenerative diseases based on clinical trials and in vivo and in vitro studies. Furthermore, they highlighted key challenges, including the low bioavailability of flavonoid-rich diets, specific therapeutic dosages, and potential adverse effects on human health. This review showcases the therapeutic potential of flavonoids in combating neurodegenerative diseases, offering a promising treatment strategy for major public health issues in an aging society.

The content and composition of bioactive compounds in various plant-based food ingredients exhibit significant variability. Additionally, certain compounds may undergo alterations during food processing, which can impact the bioavailability and bioefficacy of plant-based foods, as well as their quality characteristics, ultimately diminishing the nutritional value and acceptability of processed foods. To address this, novel or alternative food sources can be utilized to reformulate products, or biotechnological and non-traditional processing techniques can be employed to enhance the bioavailability of bioactive compounds and the nutritional value of the products. Rocío Corfield’s team (contribution 10) developed a low-energy, sugar-free snack suitable for women of childbearing age to supplement folic acid, using apple–acáchul leather as a base and incorporating folic acid. Notably, the folic acid source they used was a complex derived from folic acid and whey protein isolate, which research has shown significantly improves the bioavailability of folic acid in the product compared to the addition of folic acid alone. The total polyphenol bioavailability in this snack reaches as high as 90%. The complex formed between whey protein isolate and folic acid effectively protects the vitamin from degradation during processing and digestion, enhancing the snack’s quality and providing a new option for folic acid intake. Similarly, José Matheus (contribution 11) has innovatively designed a new trendy food by incorporating *Fucus vesiculosus* seaweeds and *Chlorella vulgaris* into hummus. This product not only offers a sustainable protein source from the algae but also alters the texture and color of the product, further enhancing its nutritional value. The new product shows a significant increase in mineral content and antioxidant activity. This innovative research presents promising solutions to the challenges of food sustainability and nutrition, offering insights for the food industry to evolve towards greater diversity and environmental responsibility. Another effective strategy for enhancing the bioavailability and nutritional value of bioactive compounds in plants during food processing is fermentation. Małgorzata Starowicz (contribution 12) and Aurelija Paulauskienė (contribution 13) have both successfully improved the nutritional value of *Brassica* vegetables and garlic processed foods through fermentation techniques. Małgorzata Starowicz conducted a systematic analysis of the dynamic changes in bioactive compounds during the fermentation of red cabbage and beetroot, revealing that the fermentation process enhances the content of phenolic compounds while reducing the levels of anthocyanins. The fermentation of these vegetables effectively diminishes the formation of advanced glycation end-products, indicating that the resulting products hold potential for the prevention of diet-related diseases. Aurelija Paulauskienė and colleagues investigated the impact of various fermentation conditions on the quality of black and white garlic. Their research demonstrated that the fermentation process significantly increased the levels of protein, fiber, vitamin C, total phenols, and antioxidant activity in garlic, while also markedly altering its color and texture. They identified the optimal fermentation process for black garlic, confirming that its chemical composition is richer than that of white garlic. Mirko Marino and others (contribution 14) thoroughly characterized the nutritional and bioactive components of freeze-dried red raspberry powder. They established that freeze-dried red raspberries are a valuable source of polyphenols, including proanthocyanidins, tannins, anthocyanins, and phenolic acids, with vitamin C and mineral content and composition being nearly identical to that of fresh red raspberries. This study addresses the gap in qualitative and quantitative research on the active components of freeze-dried red raspberries, providing foundational data for their extensive use in the food industry.

Green onions (*Allium fistulosum*), as a quintessential aromatic vegetable, are essential kitchen ingredients with diverse applications in the culinary and medicinal fields. Seong-Hoon Kim and colleagues (contribution 15) conducted a comprehensive review of the global distribution, culinary applications, nutritional significance, and therapeutic value of green onions, discussing the crucial role of incorporating green onions into the diet for maintaining overall human health. They emphasized the potential for greater application of green onions in the production of functional foods, herbal medicine, and the development of natural preservatives.

In summary, this Special Issue presents foundational data on the nutritional composition of some plant-based foods, highlighting the potential health benefits of bioactive compounds found in plants. It also offers several effective strategies for future innovation and the enhancement of plant-based foods, which are of significant relevance to researchers and food manufacturers. The papers compiled in this Special Issue contribute positively to the advancement of research in the field of plant-based foods.
